# The effect of different processing methods on nutrient and isoflavone content of soymilk obtained from six varieties of soybean grown in Rwanda

**DOI:** 10.1002/fsn3.812

**Published:** 2018-10-25

**Authors:** Marguerite Niyibituronsa, Arnold Nola Onyango, Svetlana Gaidashova, Samuel Imathiu, Mathilde Uwizerwa, Emelda Phillis Ochieng, Fredrick Ng'ang'a, Josephine Birungi, Sita Ghimire, Jagger Harvey

**Affiliations:** ^1^ Rwanda Agriculture Board Kigali Rwanda; ^2^ Jomo Kenyatta University of Agriculture and Technology Nairobi Kenya; ^3^ Biosciences Eastern and Central Africa‐International Livestock Research Institute Hub Nairobi Kenya; ^4^ Feed the Future Innovation Lab for the Reduction of Post‐Harvest Loss, and Department of Plant Pathology Kansas State University Manhattan New York

**Keywords:** chemical composition, fat, *Glycine max* (L.) Merrill, minerals, protein

## Abstract

Soymilk is rich in nutrients and isoflavones, and could greatly promote nutrition and health. However, this product is not widely accepted due to an objectionable beany flavor. Several methods involving heat treatment and soaking in basic solutions prior to soymilk extraction have been reported to reduce the objectionable flavor. However, the effects of such treatments on the nutritional value and isoflavone content of soymilk, and the responses of different soybean varieties to nutrient extraction by these methods is not well studied. The aim of this study was to determine the effect of three processing methods on protein, fat, minerals, and isoflavone content in soymilk from six soybean varieties grown in Rwanda (Peka‐6, SB 24, Sc. Sequel, Sc, Squire, and a local variety) to find the best variety and processing method. The first method (M1) involved soaking soybeans in water for 12 hr prior to milk extraction, M2 involved blanching in NaHCO_3_ prior to extraction and M3 involved soaking in NaHCO_3_ solution for 16 hr and subsequent cooking prior to extraction. M1 resulted in significantly higher nutrient and isoflavone extraction than M2 and M3. Thus, M1 extracted more nutrients and can be recommended for soymilk production. However, where consumers prefer soymilk obtained by M2 or M3, Sc Squire and the local variety may be recommended. Sc. Squire has another advantage of higher isoflavone content than the other varieties. Further comprehensive studies on the sensory acceptability of products made from different varieties by different methods among different consumer categories will be necessary.

## 1 INTRODUCTION

Soybean, *Glycine max* (L.) Merrill, is a legume belonging to the botanical family Leguminosae and subfamily of Papilionideae (Shurtleff & Aoyagi, 2007). It grows in warm temperatures with the optimum at 25°C and rainfall of 500–900 mm. Depending on maturity, soybean varieties can be early or late, being harvested within 120–130 days (Dugje et al., 2009). Globally, a total of 336.7 million metric tons of soybean were produced in 2017/2018 and the United States produced 119.52 million metric tons (USDA, 2018). In Africa, a paltry 2.1 million metric tons were produced in 2016 (FAOSTAT, 2018).

Soybean is highly nutritious: It contains about 40% protein, 20% largely unsaturated fats, 17% fiber (both soluble and insoluble), and is also a good source of the minerals such as calcium (276 mg/100 g), magnesium (280 mg/100 g), potassium (1,797 mg/100 g), iron (16 mg/100 g), and zinc (4.8 mg/100 g) (Mateos‐Aparicio, Cuenca, Villanueva‐Suárez, & Zapata‐Revilla, 2008). Its high protein is suitable for the growth of children and for management of diseases such as HIV/AIDS and tuberculosis (Gandhi, 2009). It also contains isoflavones which are associated with prevention of non‐communicable diseases (Hirose et al., 2005; Jooyandeh, 2011; Messina & Barnes, 1991; Xu, Harris, Wang, Murphy, & Hendrich, 1995).

Soybean is not cooked and eaten like other beans but is instead processed into various products for improved palatability and nutritional benefits. In Rwanda, it is mainly processed into soy flour, soymilk, and tofu (Niyibituronsa, Kyallo, Mugo, & Gaidashova, 2014). Soymilk is considered to be a cow's milk substitute for lactose intolerant individuals (Kundu, Dhankhar, & Sharma, 2018).

It is a water extract of soybeans with the following basic preparation steps: selection of soybeans, addition of water, wet grinding and separation of soymilk from fiber (okra), cooking to inactivate lipoxygenase and trypsin inhibitors, formulation and fortification (addition of sugars, salts, minerals, vitamins, flavors and thickeners to improve the nutritional and sensory attributes), and packaging (Hosken, 1999). Although soymilk could contribute to improved nutrition among rural communities in Rwanda, its acceptability is low due to the beany odor (Davles, Nlelsen, & Nielsen, 1987; Niyibituronsa et al., 2014). As a result of this, and other factors such as unavailability of high yield cultivars, soybean production in Rwanda remains low with an estimated production level of 57,089MT per year (2010) (Mugabo, Tollens, Chianu, Obi, & Vanlauwe, 2014; Nsengiyumva, Byamushana, & Rurangwa, 2017; RDB, 2015). For example, in Kamonyi District where the land under legumes is 0.18 ha/person, soybean occupies only 0.05 ha (Mujawamariya, 2012).

The beany odor of soybean occurs because of soybean lipoxygenase‐catalyzed oxidation of polyunsaturated fatty acids, mainly linoleic acid and alpha‐linolenic acid (Ediriweera, Akiyama, & Saio, 1987; Wang, Dou, Macura, Durance, & Nakai, 1997; Wilkens & Ming, 1970). A common method for soybean processing involves soaking the soybeans overnight prior to milk extraction (Hosken, 1999; Niyibituronsa et al., 2014; Nyagaya, 2008). To reduce the odor and increase acceptability of soymilk, a number of treatments or combinations of treatments have been developed for inactivation of lipoxygenase, involving heating and modification of pH (Ashraf, 1979; Kale, Pandhare, Satwase, & Goswami, 2012; Krishnan & Darly‐Kindelspire, 2013; Yan, Huan, Xin, Liang, & Shu, 2011). However, the effect of these treatments on nutrient extraction into the soymilk, and the response of different soybean varieties to nutrient extraction by these methods are not well established. The aim of this study was to determine the effect of three processing techniques on nutrient and isoflavone content of soymilk from six soybean varieties grown in Rwanda. The three techniques included (a) soaking in water prior to extraction of soymilk; (b) blanching in NaHCO_3_ solution prior to extraction; and (c) soaking with 5% NaHCO_3_ solution followed by blanching prior to soymilk extraction.

## 2 MATERIALS AND METHODS

### 2.1 Selection of soybean varieties

The six varieties selected for this study included one local variety grown in Rwanda's Western province, Peka 6 which is widely cultivated, and four varieties that are being promoted by the Rwanda Agriculture Board (RAB). The four varieties include SB 24, Sc. Saga, Sc. Sequel, and Sc. Squire, which are considered to be superior to Peka 6 in terms of early maturity, yield per unit area, and tolerance to diseases such as frog‐eye leaf spot, rust and Read leaf blotch (Rwanda Agriculture Board, 2013).

### 2.2 Sampling

Peka 6, SB 24, Sc. Saga, Sc. Sequel, and Sc. Squire samples were obtained from RAB stores in Southern and Eastern Provinces, while the local variety was obtained from farmers in the Western province where there is no RAB soybean program. Samples, 1 kg of each variety, were collected in triplicates. Samples were kept in the cold room at +4°C.

### 2.3 Soymilk preparation

Three different methods were used to prepare soymilk: soaking (M1), blanching (M2), and cooking (M3). In the first method of soaking (Hosken, 1999; Nyagaya, 2008), 25 g of soybeans were soaked in 100 ml of water for 12 hr at room temperature (25°C). After draining the soaking water and rinsing with cold water, the beans were ground with 200 ml of water using a warring laboratory electric blender (HGBTWTG4, USA) and filtered through muslin cloth. The filtrate (soymilk) was boiled in a beaker for 10 min. In the second method of blanching, 0.05 g of NaHCO_3_ and 25 g of soybean were added to 100 ml of boiling water at 100°C and blanched in a beaker for 5 min, followed by draining and rinsing with hot water. The blanching, draining, and rinsing steps were repeated twice, followed by grinding in the warring blender with 200 ml hot water (80°C) for 3 min. The mixture was filtrated with a muslin cloth followed by boiling of the filtrate in a beaker for 10 min according to the National Soybean Research Laboratory at Illinois University (NSRL, 2015). In the third method of cooking, 25 g soybean was soaked in 5% NaHCO_3_ for 16 hr at room temperature (25°C), followed by scrubbing and subsequent hot water treatment in a water bath (100°C, for 20 min). The beans were then ground with 200 ml of boiled water 98°C followed by cooking at 90°C for 10 min, homogenization, and separation of insoluble and soluble portion using muslin cloth (Kale et al., 2012).

### 2.4 Determination of nutrients content in soybean and soy milk

Soybean grains and soy milk were evaluated for protein, fat, minerals, and isoflavones. Crude protein was analyzed using a modified Folin‐Lowry Method (Lowry, Rosebrough, Farr, & Randall, 1951). Briefly, in this method, extraction was done using 5% sodium dodecylsulphate (SDS). Protein content was then measured at *λ*_nm_ = 750 using Bovine Serum Albumin (BSA) as a standard to prepare the calibration curve. Crude fat was determined using AOAC Official Method 983.23. This method involves enzymatic incubation of samples with Clarase to break down starch and subsequent extraction by blending with chloroform/methanol mixture. The aliquoted extracts are evaporated to dryness, and the total fat content determined gravimetrically. Mineral contents of the samples were determined using AOAC Official Methods in which extraction was done with concentrated nitric acid (HNO_3_) and H_2_O_2_ using a block digester (Velp Scientifica DK20/26 230V, Europe). The extracts were diluted, and determination of the target analytes calcium (Ca), magnesium (Mg), and potassium (K) was done using flame atomic absorption spectrophotometer (AAS) (GBC Savant AA 01‐1006‐03, Australia) set at wavelengths 422.70, 285.20, and 766.50 nm, respectively. Phosphorus was determined using UV‐VIS (Agilent G6860A, Australia) set at 400 nm. For quality control purposes, BCR‐708 (Institute for reference materials and measurements‐JRC European commission) was analyzed alongside the samples in determination of crude protein, oil, magnesium, calcium, and phosphorous. Isoflavones were determined using AOAC Official Method 2001.10 (Collison et al., 2008; Latimer, 2012): Extraction was done at 65°C for 2 hr in 80% methanol and the extracts saponified at ambient temperature with NaOH solution. The extracts were acidified, filtered, and diluted with 50% methanol. The extracts were then centrifuged to clarify them and analyzed by liquid chromatography on a reverse‐phase C‐18 column using HPLC (Shimadzu LC‐30A, Japan) coupled to a UV detector (SPD‐M20A, Japan) set at *λ*_nm_ = 260. The target analytes were Genistein, Genistin, Daidzin, and Daidzein. All analysis was performed in triplicates.

### 2.5 Data analysis

Data were analyzed using IBM statistics SPSS 20 software, and the analysis of variance was used to test the significance of difference between variables at 95% level of confidence. Means were separated using least significance difference (LSD) post hoc tests.

## 3 RESULTS AND DISCUSSION

### 3.1 Nutrient and isoflavone content of the six soybean varieties

**Figure 1:**
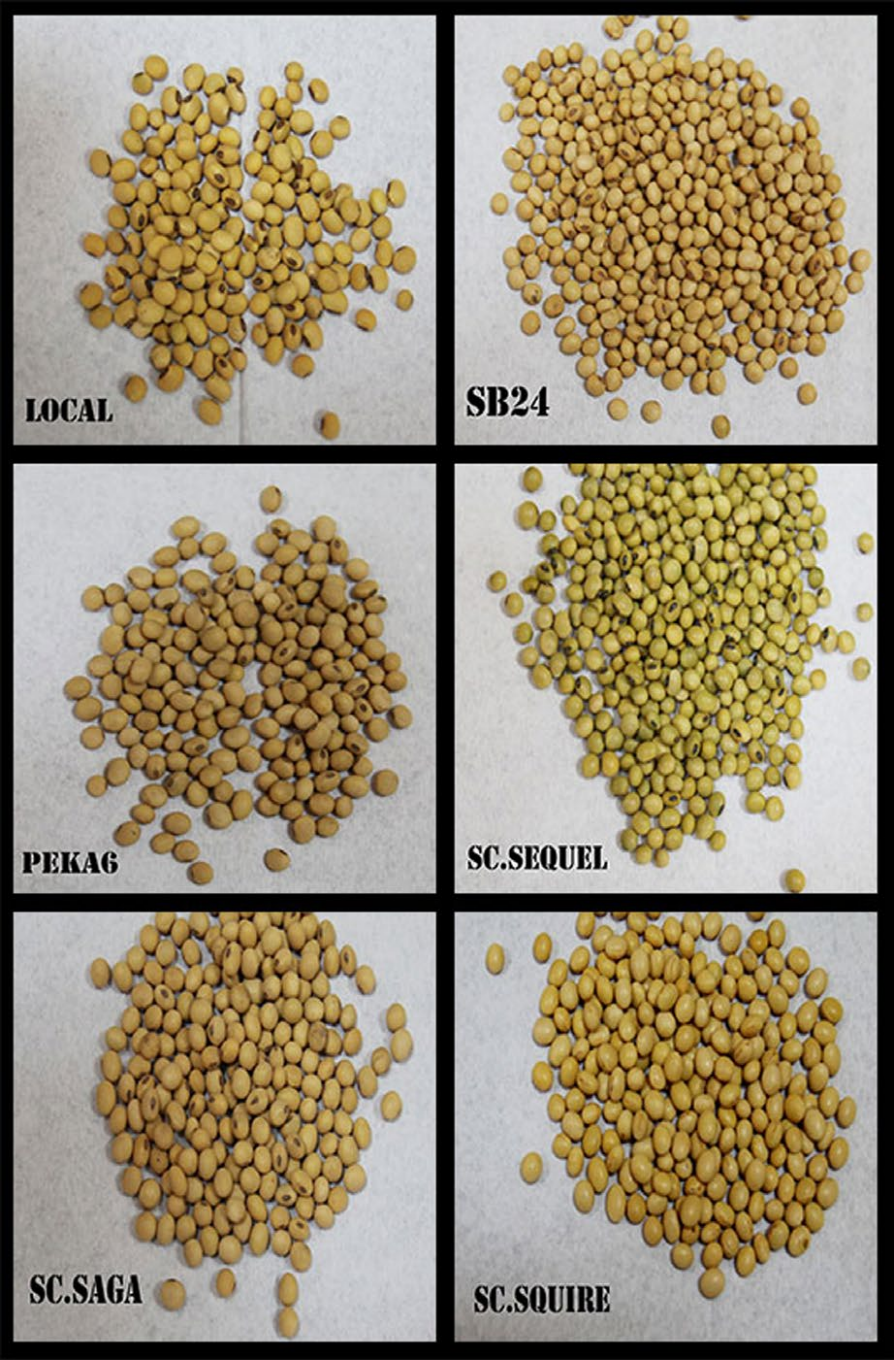
Soybean varieties used for nutrients analysis and soybean milk extraction

Local, SB24, Peka 6, and Sc. Saga have almost similar yellow grain color, as can be seen in Figure 1. The hilum is black for Local and Peka 6, and brown for SB24 and Sc. Saga. The difference is remarkable for Sc. Sequel which has a green color with black hilum and Sc. Squire which has light yellow color for both grain and hilum. According to RAB‐N2Africa report (2013), Peka 6 originated in India, SB24 in Nigeria, Sc. Saga, Sc. Squire and Sc. Sequel in Zimbabwe. The grain yield (kg/ha) was 2,406, 2,700, 3,264, 3,278, and 2,128, respectively (N2Africa, 2014).

Protein, fat, minerals, and isoflavones analyzed in six varieties of soybean grown in Rwanda are presented in Table 1. Protein content ranged between 34.7% and 36.7%, but these differences were not significant. On the other hand, there were significant differences (*p* < 0.05) in fat contents, with SB24 and Sc. Saga having the lowest (11.1%) and highest (16.6%) fat contents, respectively. These values are within the range found in previous studies (Penalvo, Matallana, & Torija, 2004). Regarding minerals, there were no significant differences in K contents, which ranged between 1451.2 and 1857.5 mg/100 g. These values are in the range of those found by Mateos‐Aparicio et al. (2008). There were no significant differences in Mg content of five of the six varieties, but the content of this mineral in Sc. Sequel (141.9 mg/100 kg) was significantly lower than the rest. There were significant differences in calcium, which ranged between 100.5 and 170 mg/kg, and phosphorus, which ranged between 318.5 and 430.9 mg/100 g. The P content of SB24 (430.9 mg/100 g) was significantly higher than the other five varieties, which on the other hand did not significantly differ from one another.

**Table 1:** Nutrients content of flour from six soybean varieties grown in Rwanda

Varieties	Protein (g/100 g)	Fat (g/100 g)	Minerals (mg/100 g)				Isoflavones total (μg/g)
			Ca	Mg	K	P	
Peka 6	36.7 ± 4.4^a^	12.4 ± 0.9^b^	131.7 ± 12.2^b^	164.9 ± 12.8^a^	1453.5 ± 299.1^a^	325.5 ± 60.9^b^	2193.5 ± 196.6^b^
Sc. Saga	34.7 ± 2.3^a^	16.6 ± 2.3^a^	148.2 ± 24.8^a^	166.4 ± 15.7^a^	1701.9 ± 300.9^a^	318.5 ± 72.9^b^	1942.6 ± 591.4^b^
Sc. Sequel	36.1 ± 5.4^a^	14.9 ± 1.9^ac^	170.5 ± 47.5^a^	141.9 ± 11.2^b^	1451.2 ± 315.5^a^	360.7 ± 108.7^b^	1666.6^bd^ ± 346.5
Sc. Squire	36.0 ± 3.5^a^	15.6 ± 1.2^a^	120.7 ± 8.5^b^	150.1 ± 10.9^a^	1857.5 ± 371.3^a^	426.1 ± 51.2^b^	4839.9 ± 375.3^a^
SB24	36.4 ± 1.3^a^	11.1 ± 0.7^b^	100.5 ± 11.8^b^	167.2 ± 31.9^a^	1625.4 ± 343.9^a^	430.9 ± 50.3^a^	2272.3 ± 341.3^b^
Local	36.7 ± 2.0^a^	12.7 ± 0.7^bc^	144.4 ± 3.3^a^	154.0 ± 9.5^a^	1669.8 ± 6.1^a^	414.9 ± 3.5^b^	2528.6 ± 444.9^bc^

Data are expressed as means ± *SD*. Means within the column with the same superscript letter are not significantly different.

The isoflavones content was significantly different between varieties (*p* < 0.05). Sc. Squire had the double content of isoflavones 4839.9 μg/g compared to other varieties, while Sc. Sequel had the lowest (1666.6 μg/g). The values are higher than those reported for Korean varieties, where the values ranged between 1,248 and 1,528 mg/Kg (Yong‐Soon, Bung‐Hoon, Jong‐Hwa, & Nam‐Soo, 2000).

### 3.2 Nutrient and isoflavone content of soymilk processed by different methods

Table 2 shows the nutrient and isoflavone content of soy milk obtained by the three different methods (M1, M2, and M3) from a blend of soybeans from all the six varieties. The nutrients (proteins, fat, minerals) and isoflavones (daidzin, genistin, genistein, and daidzein) were better extracted by M1 than M2 and M3. Remarkably, the amount of the isoflavones daidzein and genistein extracted by M1 (9.8 and 9.4 μg/ml, respectively), were between 4 times and 10 times higher than M2 and M3 (Table 2). This is consistent with a previous report that soaking or heating led to a 12% or 49% loss, respectively, in soy isoflavones during tempeh processing, and that alkaline extraction caused a 53% loss during soy protein isolate production (Wang & Murphy, 1996). The more the bioactive isoflavones (daidzein and genistein), the more the health benefits (Setchel & Cassidy, 1999).

**Table 2:** Effect of different methods on nutrient content of soy milk prepared from a blend of soybean from the six varieties

Nutrients means	Method 1	Method 2	Method 3	*p*‐value
Protein (g/100 g)	3.4 ± 0.3^a^	2.3 ± 0.7^b^	2.1 ± 0.2^b^	<0.05
Fat (g/100 g)	1.7 ± 0.2^a^	1.1 ± 0.4^b^	1.5 ± 0.2^a^	<0.05
Minerals (mg/100 g)				
Ca	9.0 ± 2.3^a^	6.9 ± 1.7^b^	5.4 ± 1.4^b^	<0.05
Mg	13.6 ± 1.7^a^	10.9 ± 1.9^b^	8.1 ± 0.8^c^	<0.05
K	146.9 ± 30.9^a^	116.1 ± 27.4^b^	98.6 ± 15.9^b^	<0.05
P	26.4 ± 8.6^a^	18.9 ± 7.3^b^	18.3 ± 5.8^b^	<0.05
Isoflavones				
Daidzin	71.6 ± 24.1^a^	61.1 ± 19.7^a^	58.8 ± 25.4^a^	>0.05
Genistin	80.6 ± 25.8^a^	55.7 ± 19.3^b^	62.1 ± 12.4^c^	<0.05
Daidzein	9.8 ± 3.4^a^	1.6 ± 0.5^b^	2.1 ± 1.2^b^	<0.05
Genistein	9.4 ± 4.2^a^	0.6 ± 0.4^b^	1.0 ± 0.3^b^	<0.05
Total isoflavones (μg/ml)	171.3 ± 53.2^a^	119.0 ± 38.1^b^	124.1 ± 48.4^b^	<0.05

Data are expressed as means ± *SD*. Means within the row with the same superscript letter are not significantly different. (Method 1, soaking grains for 12 hr; Method 3, blanching grains; Method 3, cooking grains).

The reduced extraction of nutrients and isoflavones by M2 and M3 might be due to a common factor. Unlike M1, these two methods involve heating prior to extraction. Heating denatures proteins, and may reduce their extraction due to decreased solubility (Nufer, Ismail, & Hayes, 2009; Zhang, Guo, Liu, & Chang, 2012). Protein denaturation also affects isoflavone thermal stability (Malaypally & Ismail, 2010; Zhang, Chang, & Liu, 2015). Many plant proteins are not readily soluble in water due to hydrophobic residues and S‐S linkages, and alkaline solutions are often used to increase their solubility through breakage of hydrogen bonds (Cui et al., 2017). However, M3, involving soaking in NaHCO3 solution for 3 hr followed by heating had lower protein extraction than M1, indicating that the effect of heating on reducing protein extraction is stronger than the alkaline treatment‐mediated increase in protein extraction. No differences were observed between M2 and M3 for protein, Calcium, and Phosphorous extraction. On the other hand, M2 extracted more magnesium than M3, and the reason for this difference is unclear.

### 3.3 Nutrient content of soymilk prepared from six soybean varieties by three different methods

**Figure 2:**
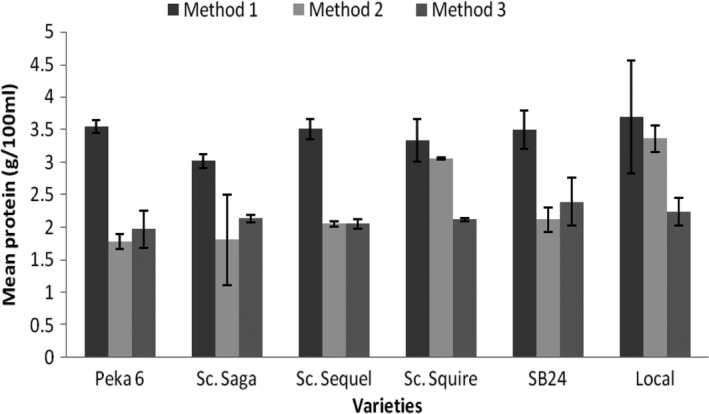
Comparison of means for protein content of soymilk per variety and method (Method 1, soaking grains for 12 hr; Method 3, blanching grains; Method 3, cooking grains)

Although M1 was found in this study to give better nutrient extraction than M2 and M3, the latter two methods were developed for reducing beany flavor, and thus some people might prefer milk processed by M2 or M3 rather than M1. Moreover, at least in one study, it was shown that the efficiency of extraction of solids from soybeans into soymilk is dependent on the varieties (Zhang et al., 2015). Thus, it was of interest to determine whether M2 and M3 might have better nutrient extraction from some of the varieties. As shown in Figure 2, M1 produced soymilk with higher protein content in all the varieties. However, for Sc. Squire and the local variety, M2 gave protein content comparable to M1. This might be related to the texture inside the grain as observed during soybean milk preparation; the two varieties are relatively softer and tender.

**Figure 3:**
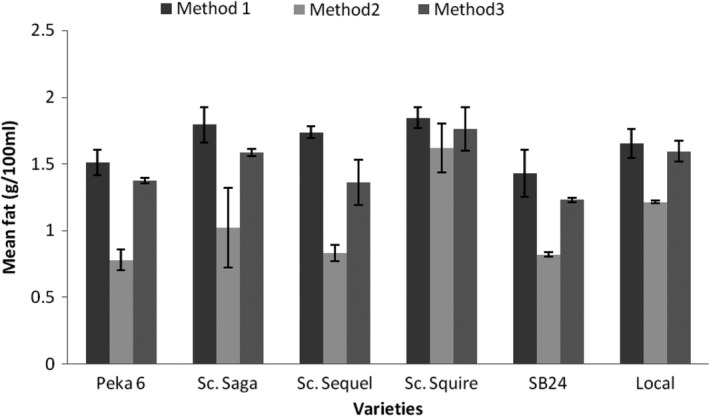
Comparison of means for fat content of soymilk per variety and method (Method 1, soaking grains for 12 hr; Method 3, blanching grains; Method 3, cooking grains)

Figure 3 shows that M2 gave the lowest fat content in all the varieties and that, for Sc. Squire and the Local variety, M3 gave fat contents comparable to M1. The lower fat extraction by M2 is consistent with a previous finding that increasing temperature in the range 100–150°C led to a reduction in fat extraction (Adejumo, Alakowe, & Obi, 2013; Saldaña & Martínez–Monteagudo, 2013).

**Figure 4:**
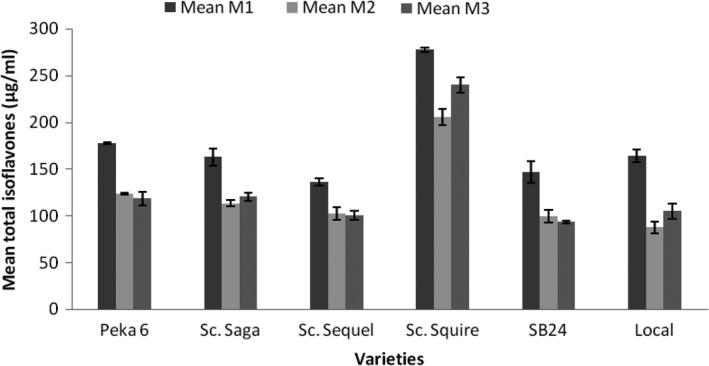
Comparison of means for total isoflavones content of soymilk per variety and method (Method 1, soaking grains for 12 hr; Method 3, blanching grains; Method 3, cooking grains)

As shown in Figure 4, isoflavones were also better extracted by M1 than M2 and M3 in all the varieties. This is consistent with the study done previously showing that soymilk produced at strong heat had lower total isoflavones (Toda, Sakamoto, Takayanagi, & Yokotsuka, 2000).

Neither M2 nor M3 afforded comparable isoflavone extraction as M1 in any of the varieties. Soy milk from variety Sc. Squire had much higher isoflavones content than the rest of the varieties. Thus, Sc. Squire Soymilk with the lowest isoflavone content, obtained by M2, had higher isoflavone content than soymilks obtained from the other varieties, by even M1.

## 4 CONCLUSION

Soymilk processing by a method involving soaking for 12 hr prior to extraction and subsequent pasteurization afforded better extraction of nutrients and isoflavones than methods involving heating prior to extraction. Sc. Squire and the Local variety exhibited higher extraction of proteins and fat than the other varieties during soy milk processing by the methods involving heating prior to extraction. Thus, these two varieties, and especially Sc Squire that has higher isoflavone content than the rest may be recommended for soymilk preparation whenever M2 or M3 is used. However, future comprehensive studies on the sensory acceptability of products made from different varieties by different methods among different consumer categories will be necessary.

## CONFLICT OF INTEREST

None.

## ETHICAL STATEMENT

This study does not involve any human or animal testing.
